# Screening for compensated advanced chronic liver disease using transient elastography in outpatient addiction clinics

**DOI:** 10.1111/acer.15463

**Published:** 2024-10-13

**Authors:** Antoine Karrer, Raphael Pangui, Caroline Le Lan, Sebastien Le Texier, Antonia Le Gruyer, Florence Moirand, Theophile Chalvin, Romain Moirand

**Affiliations:** ^1^ Service des maladies du foie et de l'appareil digestif, CHU Pontchaillou Rennes France; ^2^ Addictologie de Liaison, CHU Rennes, et Centre Hospitalier Guillaume Reignier, Pôle Addiction Précarité Rennes France; ^3^ CHU Rennes, Service des Maladies du Foie et UF Addictologie Rennes France; ^4^ Centre Hospitalier Guillaume Reignier, Pôle Addiction Précarité Rennes France; ^5^ Yves Le Foll Hospital, Service de gastro‐entérologie et addictologie Saint Brieuc France; ^6^ Addiction France, Centre de Soins d'Accompagnement et de Prévention en Addictologie Saint Brieuc France; ^7^ Univ Rennes, INRAE, INSERM, CHU Rennes, Institut NUMECAN (Nutrition Metabolism and Cancer), Service des Maladies du Foie et UF Addictologie Rennes France

**Keywords:** addiction clinics, compensated advanced chronic liver disease, screening, transient elastography

## Abstract

**Background:**

Patients with substance use disorders present with many risk factors for liver disease—including alcohol, hepatitis C virus infection, and obesity—and should thus be screened for compensated advanced chronic liver disease (cACLD). Such screening could potentially be performed by outpatient addiction clinics. In this study, we aimed to assess the feasibility, acceptability, and results of cACLD screening using transient elastography (TE) among all patients attending routine follow‐up visits at addiction clinics, regardless of their liver disease risk factors.

**Methods:**

Liver fibrosis evaluation using TE was offered to every patient consulting two different addiction clinics in France, between December 2020 and September 2021, during dedicated half‐day screening sessions. The screening was proposed during the patient's routine care and was performed immediately after the scheduled consultation. Patients with a liver stiffness measurement over 8 kPa were referred to a hepatology visit in the addiction clinic within 2–4 weeks.

**Results:**

Screening was offered to 227 patients and was accepted by 116 (51%) patients. Twelve patients had a liver stiffness over 8 kPa, and nine of these patients attended the recommended specialist hepatology visit. Five patients (4.3% of those screened) were diagnosed with cACLD. Patients' acceptance of the screening was associated with older age, being on one's own or professionally inactive, and presenting with alcohol use disorder.

**Conclusion:**

Overall, our results demonstrated that opportunistic cACLD screening using TE in outpatient addiction clinics was feasible and acceptable, with good results.

## Graphical abstract

Patients visiting two outpatient addiction clinics were offered liver stiffness measurement (LSM) during 13 dedicated half‐day screening sessions. Half of them accepted. 10% had LSM > 8 kPa, and a hepatology visit was organized in the clinic within 2–4 weeks. 4.3% were classified as having compensated advanced chronic liver disease (cACLD). Patients offered the procedure represented 21.4% of the patients attending the clinic during a 6‐month period. Opportunistic cACLD screening at an addiction clinic is acceptable and feasible in terms of staff involvement.

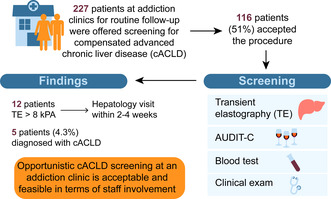

## INTRODUCTION

Liver disease is an important public health concern worldwide. Chronic liver diseases are asymptomatic for years, and progress from no fibrosis to severe fibrosis, and then compensated cirrhosis, before the occurrence of severe complications. The term compensated advanced chronic liver disease (cACLD) describes asymptomatic patients with either severe fibrosis or compensated cirrhosis, who are difficult to clinically identify (de Franchis, 2015). This concept emerged from the availability of noninvasive diagnostic methods, such as liver stiffness measurement (LSM) by transient elastography (TE), which shows very good performance in distinguishing between no or mild fibrosis and cACLD in most liver diseases (Singh et al., 2017), including alcohol‐related liver disease (ALD) (Legros et al., 2022). When a patient is identified as having cACLD, a number of actions can be taken to improve prognosis, including removal or suppression of the primary etiological factor and other liver disease risk factors, treatment with nonselective β‐blockers, screening for hepatocellular carcinoma, and vaccinations (de Franchis et al., 2022). Thus, it is important to screen for cACLD in populations at risk of hepatic fibrosis.

In France, as in numerous other countries, the main causes of liver disease are alcohol consumption, hepatitis C virus (HCV) infection, and obesity/metabolic syndrome. Recent French guidelines propose the use of TE to screen at‐risk alcohol drinkers over 40–45 years of age (Louvet et al., 2022). Patients with HCV infection should also benefit from cACLD screening (Leroy et al., 2021; Tran et al., 2022). Notably, obesity is frequently associated with past or current at‐risk alcohol use (Lewis, 2020), sometimes through an addiction transfer (Junghanns et al., 2000).

Patients with substance use disorders often receive outpatient care at addiction clinics, which could thus be appropriate settings for cACLD screening. However, medical follow‐up can be a challenge in this population, and it is unclear whether such a screening would be considered acceptable by those attending outpatient clinics. Prospective cohort studies have been conducted among people who inject drugs (PWID) to determine the acceptability and performance of using TE to screen for viral cACLD in community settings, including outpatient addiction clinics (Foucher et al., 2009; Marshall et al., 2015; van Santen et al., 2018). The feasibility of such screening was also explored in a community alcohol support setting in Edinburgh, excluding patients without alcohol history (Matthews et al., 2019), and a feasibility randomized control trial tested the possibility to influence alcohol consumption behavior with advice tailored on LSM in patients with alcohol problem (Subhani et al., 2023). These studies yielded encouraging results, but did not present data regarding the screened population/target population ratio, and each focused on only one liver disease risk factor.

In the present study, we aimed to determine the feasibility, acceptability, and results of a general cACLD screening using TE, offered to all patients attending an outpatient addiction clinic, regardless of their liver disease risk factors.

## PATIENTS AND METHODS

### Time of inclusion

This prospective cohort study was conducted in an outpatient addiction clinic in Rennes, France, with an inclusion period from November 2020 to May 2021, and in another outpatient addiction clinic in Saint Brieuc, France, from May to November 2021. Evaluation by TE was offered to each patient who attended the clinic for a follow‐up visit during one of the dedicated half‐day screening sessions. In total, there were 13 screening sessions at the first clinic—covering the whole week, from Monday morning to Friday afternoon, because patients attending regular follow‐up usually come on a specific day of the week—and there were 3 screening sessions at the second clinic.

### Protocol

#### Screening session

Patients were not informed in advance about the scheduled offer of screening. At the first clinic, screening was most often proposed by the healthcare professional (doctor, nurse, social worker, psychologist, or dietician) who received the patient. Screening was less commonly proposed in the waiting room by the investigator (AK, a medical intern specializing in hepatology and addiction medicine). A billboard explaining and offering the screening was installed in the waiting room and was removed at the end of the session. At the second clinic, screening was always proposed by the investigator in the waiting room.

Among the patients who were offered the screening and refused it, all agreed to be included in this study as “refusers.” The reason for refusal was not collected. Patients who accepted the screening were seen immediately after their visit by the investigator, who provided oral and written information. No patients refused to participate at this point. Next, patient data were collected, and then TE was performed. Fasting was not a prerequisite.

#### Hepatology visit

Patient exhibiting an LSM over 8 kPa were advised to attend a hepatology visit at the clinic within 2–4 weeks, with the same investigator. Patients who accepted were given a prescription to undergo a blood test 2 days before the visit. Patients were told to come to the visit after fasting for 4 h. If patients missed their first visit, they were contacted and a second visit was proposed. During this visit, a detailed medical interview and a physical examination were performed. The investigator also repeated TE. The duration of fasting before the visit was recorded.

### Procedures

TE was performed by the investigator, using Fibroscan® mini 430+ (Echosens, Paris), with an M probe, following the manufacturer's recommendations. TE was considered valid if at least 10 measures were performed, and if the interquartile range (IQR) was <30% of the median, or if the median was <7.1 kPa.

The data collected during the screening session included the patient's age, sex, average alcohol consumption in the past 7 days (grams per day), motivation for alcohol cessation using a visual analogy scale (VAS), craving for alcohol consumption using a VAS, and the Alcohol Use Disorders Identification Test Consumption (AUDIT‐C) (Higgins‐Biddle & Babor, 2018).

The data collected during the hepatology visit included the patient's cardiovascular risk factors; quantification, rhythm, and duration of alcohol consumption; quantification, rhythm, and duration of tobacco and cannabis consumption; average alcohol consumption in the past 7 days; motivation for alcohol cessation using a VAS; and craving for alcohol consumption using a VAS. Clinical examination was performed to assess for signs of metabolic syndrome and cirrhosis.

Blood tests were performed only among patients attending the hepatology visit and included cell blood count (CBC) with platelets; aspartate aminotransferase (AST); alanine aminotransferase (ALT); gamma‐glutamyl transpeptidase (GGT); alkaline phosphatases (PAL); bilirubin; prothrombin time (PT); lipid profile and fasting blood sugar (FBS), if not assessed within the last 3 months; and HCV, hepatitis B virus (HCB), and human immunodeficiency virus (HIV) serologies, if not evaluated within the last 12 months.

Patients were finally classified as having cCALD based on the LSM performed during the hepatology visit. We applied the 10‐kPa cut‐off that Rasmussen et al. (2021) showed to be accurate for prognosis.

For all patients who were offered the screening at the first clinic, the following data were retrospectively collected from the electronic patient file: housing, relatives, occupation, income, follow‐up duration, psychiatric history, history of incarceration, opioid substitution therapy prescription, main substance that led to addiction care, and alcohol abstinence duration. The total number of patients who attended the first clinic at least once during the inclusion period was obtained from the clinic's information system.

### Ethical considerations

The protocol was approved by the ethics board of Centre Hospitalier Universitaire de Rennes (decision number 21‐45). The study was categorized as a “routine care study” because screening for cACLD by TE is already recommended. Each participant was delivered written and oral study information and gave no opposition prior to participation.

### Statistical analyses

The primary outcome was the proportion of patients who accepted TE. Secondary outcomes included the proportion of the target population (patients who attended at least one visit to the first addiction clinic during the study period) who were offered screening and the proportion who accepted; the proportion of patients who attended the hepatology visit when exhibiting an LSM over 8 kPa; the comparison of demographic and addiction‐related data between patients who accepted versus refused TE; and difference in average alcohol consumption during the past 7 days, craving for alcohol, and motivation for alcohol cessation between the first TE screening and the hepatology visit.

The results are expressed as mean ± SD, median [IQR] or number (percentage). Qualitative variables were compared using the chi‐square test or Fisher's exact test, as appropriate. Quantitative variables were assessed using Student's *t* tests. We performed univariate analysis using logistic regression models to estimate the associations between screening acceptance (yes/no) and demographic and psychosocial variables in patients from the first clinic. To adjust for various covariates, we used a multivariate logistic model with mixed (forward/backward) stepwise selection, including all variables that were associated with acceptance in univariate analyses (*p* < 0.10). Statistical analyses were not performed for the evolution of average alcohol consumption in the past 7 days, craving for alcohol, and motivation for alcohol cessation between the TE screening and hepatology visit, because the number of patients was insufficient. All statistical analyses were performed using JMP pro 16 (the SAS institute, Cary, USA).

## RESULTS

**FIGURE 1:**
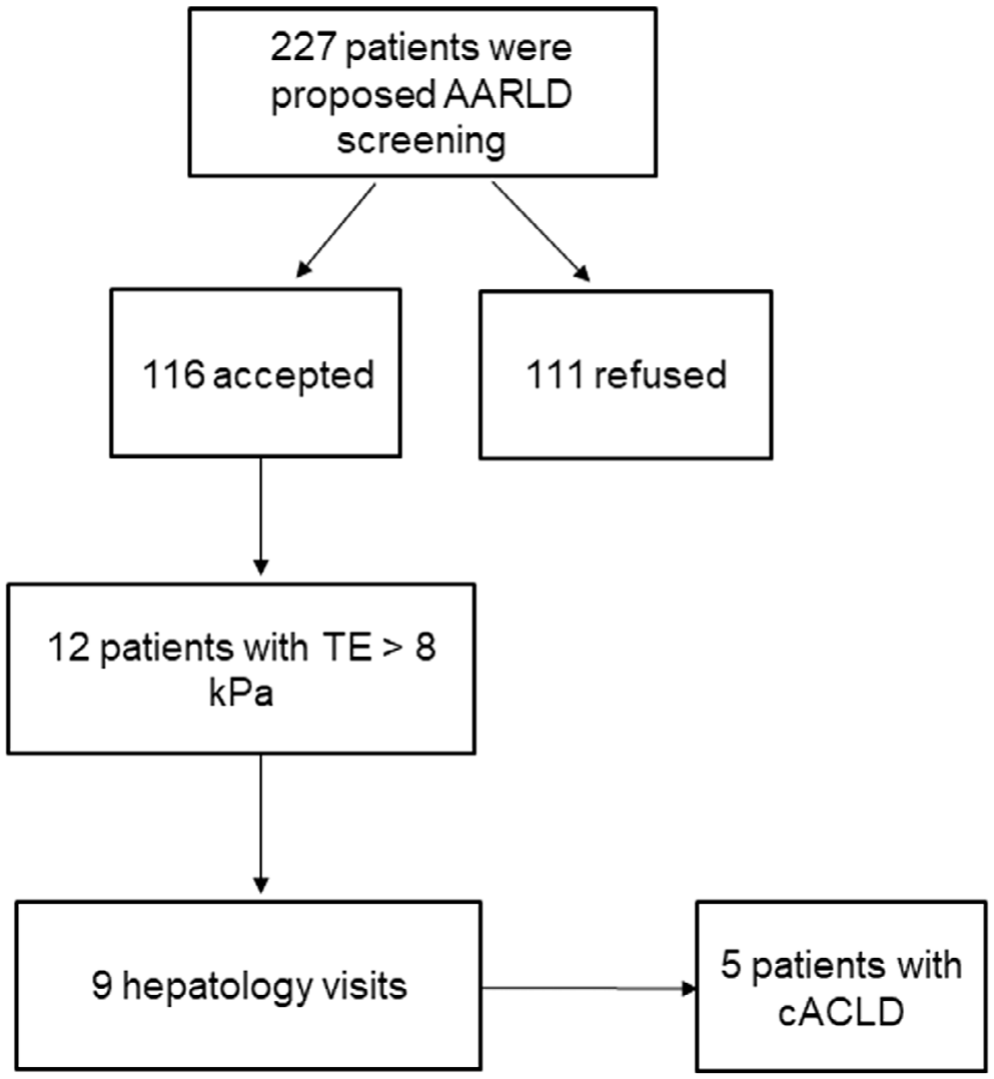
Patient flow chart.

The screening procedure was offered to 227 patients at the two addiction clinics: 208 patients at the first clinic, and 19 at the second clinic. Among these 227 patients, 116 accepted the screening and 111 refused, yielding a 51% acceptability rate. Figure 1 shows the patient flow chart.

At the first clinic, 971 different patients attended at least one visit with at least one healthcare professional between November 2020 and May 2021, which corresponds to 127 full days of opening. During 13 half‐day sessions, TE screening was proposed to 208 patients during this period, of whom 102 accepted. Therefore, screening was proposed to 21.4% of the potential beneficiaries, and 10.6% were screened.

Among the 116 screened patients, 12 (10.6%) exhibited an LSM over 8 kPa. Of these 12 patients with an elevated LSM, 9 (80%) attended the recommended hepatology visit. Five of these patients (4.3% of the screened patients) were definitively diagnosed with cACLD.

Table 1 summarizes the patients' demographic and addiction‐related characteristics. The mean age was 40.3 years, and the sex ratio was slightly in favor of men. About half of the patients were frequently on their own (47.4%) and were not employed (53%). The majority of patients were attending the clinic mainly for alcohol use disorder (77.2%), and few were abstinent from alcohol use (13.4%). A small proportion of patients were receiving opioid substitution therapy (5.2%). Around one‐fifth of patients had been in prison (19.3%) or presented with psychiatric history (20%).

**TABLE 1:** Patients' characteristics according to acceptation or refusal of screening procedure.

	All	Acceptation	Refusal	*p* ^a^	RR [IC95%]^b^	*p* ^b^
*N* (%)	227	116 (51)	111 (49)			
Age, mean ± SD	40.3 ± 12.3	43.5 ± 11.5	37.8 ± 12.5	0.001	1.03 [1.01–1.06]	0.009
Sex, *n* (%)						
Male	138 (61)	76 (65)	62 (56)	NS		
Female	85 (39)	40 (35)	45 (44)			
Relatives, *n* (%)^c^, living with						
Alone	97 (47)	56 (55)	41 (37)	0.007^d^	1.81 [1.01–3.24]	0.46^d^
Friend	8 (4)	2 (2)	6 (5)			
Spouse	25 (12)	11 (11)	14 (13)			
Children	22 (11)	10 (10)	10 (9)			
Spouse + children	24 (12)	10 (10)	14 (13)			
Parents	25 (12)	5 (5)	18 (16)			
Other	6 (3)	4 (4)	2 (2)			
Incomes, *n* (%)^c^						
Employment	91 (47)	36 (35)	55 (50)	0.01^e^	0.50[0.28‐0.89]	0.018^e^
Allowance for disabled adults	36 (17)	19 (18)	13 (12)			
Social support	18 (9)	10 (10)	8 (7)			
Pension	16 (8)	12 (12)	4 (4)			
Unemployment insurance	14 (7)	9 (9)	5 (5)			
Other	22 (11)	9 (9)	13 (12)			
Occupation, *n* (%)^c^						
Full‐time	87 (44)	35 (34)	52 (47)	0.002^f^	–	
Part‐time	16 (8)	10 (10)	6 (5)			
Student	15 (8)	4 (4)	11 (10)			
Unemployed	31 (16)	17 (17)	14 (13)			
Retired	8 (4)	4 (4)	4 (4)			
Other inactive	41 (21)	29 (28)	12 (12)			
Main substance of abuse, *n* (%)^c^						
Alcohol	153 (77)	82 (80)	71 (64)	0.02^g^	–	
Cannabis	26 (13)	8 (8)	18 (16)			
Other	19 (10)	7 (7)	12 (11)			
Alcohol abstinence, *n* (%)^c^	20 (13)	8 (8)	12 (11)	NS		
Opioid substitution therapy, *n* (%)^c^	8 (6)	6 (6)	2 (2)	NS		
Incarceration history, *n* (%)^c^	25 (19)	11 (11)	14 (13)	NS		
Psychiatric history, *n* (%)^c^	42 (20)	23 (23)	19 (17)	NS		
TE > 8 kPa, *n* (%)	–	12 (10)	–	–		

^a^ *p* value in univariate analysis.

^b^ Relative risk (RR) and confidence interval (CI), and *p* value after multivariate logistic regression.

^c^ Analysis restricted to 208 patients from clinic 1.

^d^ Comparison between patients living alone versus living with relative.

^e^ Comparison between employed versus other patients.

^f^ Comparison between active patients (employed or students) versus non active patients.

^g^ Comparison between patients consulting for alcohol use disorder versus other substances.

Table 1 also compares the characteristic of the patients who accepted versus refused TE at the first clinic. Those who accepted TE were older (43.5 years vs. 37.8 years; *p* = 0.001), more frequently on their own (61% vs. 38%; *p* = 0.007), less likely to get money from a job (39% vs. 61%; *p* = 0.01), more frequently professionally inactive (62% vs. 38%; *p* = 0.002), and more commonly attending the clinic for alcohol use disorder than for other substances (54% vs. 46%; *p* = 0.02). After logistic regression, only age, living alone or not, and income remained significant in the model.

Table 2 presents additional data regarding alcohol consumption, only for patients who accepted screening. Mean alcohol consumption during the past week showed important variation among patients, with some being abstinent for a long period and others reporting very heavy consumption.

**TABLE 2:** Alcohol consumption among patients who accepted the screening procedure.

AUDIT C	
Drinking occasions frequency	4 [2–4]
Number of drinks on a typical day	3 [1–4]
Heavy drinking occasions frequency	3 [2–4]
Total score	9 [6–12]
Craving intensity (on VAS)	4.5 ± 3.2
Motivation to quit (on VAS)	7.1 ± 3.0
Alcohol in g/day in the seven last days	45.5 ± 66.3

*Note*: AUDIT C: drinking occasions: never (0); monthly or less (1); 2–4 times a month (2); 2–3 times a week (3); 4 or more times a week (4). Number of drinks on a typical day: 1–2 (0); 3–4 (1); 5–6 (2); 7–9 (3); 10 or more (4). Heavy drinking occasions: never (0); less than monthly (1); monthly (2); weekly (3); daily or almost daily (4). Results presented as median [IQR] or mean ± SD.

Tables S1 and S2 summarize the data collected during the hepatology visit. Patients frequently exhibited metabolic comorbidities and were older than the rest of the population: 56 [48–61] years vs. 42 [35–48] years (*p* < 0.001). No participants had serological markers for hepatitis B or C viruses or HIV. At the end of the evaluation, five patients were definitively diagnosed with cACLD. Three other patients exhibited only marginally increased elastography at screening and slightly decreased elastography during the hepatology visit. This could be partly explained by fasting before the second measurement. It is less likely to be an effect due to decreased inflammation, because there was not a significant reduction of alcohol consumption between the two LSM time‐points. One patient exhibited an important discordance between the two TE measures. Table S3 summarizes all the data related to alcohol variables variations between the first TE screening and the hepatology visit, which did not show significant variations.

## DISCUSSION

The results of this prospective study showed a 51% acceptability rate of an opportunistic screening procedure for cACLD, involving proposal of immediate TE during a routine visit at an addiction center. Factors associated with screening acceptance were age, living on one's own, and being professionally inactive. Among the patients found to have elevated LSM on TE, 80% attended the recommended hepatology visit. Overall, 4.3% of the screened patients were diagnosed with cACLD.

We did not focus on any specific etiology of liver disease, considering that patients, especially at addiction clinics, often have many risk factors. In contrast, previous studies have focused on one cause, mainly HCV infection in the context of drug injection. Foucher et al. (2009) offered TE screening to drug users in two street‐based outreaches, and reported a very high acceptance rate, and found that 20% of patients had elastography greater than 7.1 kPa, and 4.7% greater than 12.5. However, they provided no information regarding the rate of hepatology visits according to fibrosis. A study in a similar population from Australia tested a quite different intervention campaign to promote noninvasive fibrosis assessment—which included recruitment posters, an educational resource package, and a financial incentive—but did not report the acceptability rate (Marshall et al., 2015). In a more recent study, Matthews et al. (2019) focused on alcohol‐related liver disease (ALD) in a community alcohol support service. Their study was advertised on a rolling TV screen and posters, and potential participants could request a specific appointment for TE. They reported a 67% rate of uptake among those who requested information, but did not provide the total number of patients who were followed by the service.

In our study, alcohol use disorder was the first diagnosis in most patients, and the proportion of PWID was low. This was because another clinic in the same town is the primary clinic devoted to substitutive treatment for opioid use disorder. Compared to a report on the global activity of outpatient addiction clinics in France (Palle, 2021), our patients more frequently consulted for alcohol use disorder rather than for other substances (77.2% vs. 51.3%), were more often on their own (47.3% vs. 33.2%), and had a more balanced sex ratio (56.3% male vs. 77.2% male). On the other hand, our patients were similar in mean age (40.3 vs. 40.4 years) and in other characteristics, such as the rates of penal‐imposed care, psychiatric history, and homelessness.

An interesting finding of our study was that the protocol allowed for a single investigator to propose screening to 20% of the whole population attending the clinic during 6 months, in only 13 half‐days. Given the implication of cACLD diagnosis, this workload seems quite acceptable, especially since the screening procedure can be performed by trained nurses. The factors associated with screening acceptance—being older, and having no relatives or professional obligations—suggest that acceptance of screening was largely determined by a patient's immediate availability to stay longer after their visit to attend the TE screening. Thus, the acceptance rate might be improved by proposing, in cases of refusal, a specific screening visit during a subsequent screening session. We observed good rates of attendance to the hepatology visit, and acceptability of the complementary blood tests, as reported in other studies (Matthews et al., 2019). This may have been partly influenced by the fact that the hepatology visit took place at the addiction clinic.

The 4.3% rate of cACLD detected in the present study must be interpreted cautiously, due to the number of subjects. It was lower than in other studies that have focused on PWID with a high HCV infection prevalence (Marshall et al., 2015; van Santen et al., 2018). This rate could be also influenced by the relatively young age of the population. However, although cACLD is usually observed in older patients, hepatic decompensation is occasionally observed in younger patients with heavy alcohol consumption, such as those seen in addiction clinics. Thus, it is difficult to recommend focusing on any certain age group in that specific population. Indeed, the observed prevalence represents a good result for a screening procedure, meaning that the population encountered in an outpatient addiction clinic could be a suitable target. Notably, it will be necessary to repeat the screening procedure, although the optimal frequency remains to be determined.

One interest in screening for cACLD is that it could motivate patients to reduce alcohol consumption. Indeed, a meta‐analysis showed a significant association between adding advice based on biomarkers of liver injury (mainly blood tests) to routine care and a reduction of harmful alcohol consumption (Subhani et al., 2021). More recently, two studies indicated a positive effect of screening procedure based on LSM on self‐reported alcohol intake (Kjaergaard et al., 2024; Subhani et al., 2023). We did not identify any such effect during the short interval between screening and the hepatology visit, as there were no noticeable differences in the three parameters (evolution of average alcohol consumption, craving for alcohol, and motivation for alcohol cessation) that were evaluated between these two visits.

This study had several limitations. The most important is that the reasons for screening refusal were not collected; thus, we cannot ascertain whether these patients would have accepted a screening visit at a subsequent session. This study was prospective; however, most of the data shown in Table 1 were retrospectively collected from the patients' files in the first clinic. Third, the choice of the 8‐kPa threshold for the TE measurement is questionable. The Baveno VII consensus recommends a TE measurement threshold of ≤10 kPa to exclude cACLD (de Franchis et al., 2022). This threshold has been demonstrated to have important prognostic value (Rasmussen et al., 2021). We chose an 8‐kPa threshold for use at the first visit to increase the sensitivity of screening, but applied a threshold of 10 kPa at the second LSM for final classification. Finally, this study was conducted during the COVID pandemic and included two confinement periods in France—from October 30th to December 15th in 2020 and from April 3rd to May 3rd in 2021. However, restrictions during these two periods were much less severe than during the first confinement starting in March 2020, and the two clinics were operating quasi‐normally, with only a limitation of the number of people in the waiting room and mask obligation. Notably, in a study at another outpatient clinic, we showed that during the third wave, patients felt that the COVID‐19 crisis led to only minor disruptions in access to healthcare (Constant et al., 2022).

In conclusion, the present study demonstrated that opportunistic cACLD screening using TE, during a routine visit at an addiction clinic, was feasible and acceptable. This also appears to be a good method for initiating specialized hepatology follow‐up in cases with increased TE measurement. Moreover, it seems feasible to screen most of the cohort of a clinic, in terms of staff involvement, especially if a hepatology consultant is available at the clinic for patients with increased TE.

## CONFLICT OF INTEREST STATEMENT

No conflict of interest.

## Supporting information


TABLES S1–S3.

## Data Availability

The data that support the findings of this study are available from the corresponding author upon reasonable request.
