# Supplementary material for: Screening for compensated advanced chronic liver disease using transient elastography in outpatient addiction clinics
Source: Alcohol Clin Exp Res (Hoboken). 2024 Oct 13;48(12):2303–9. doi: 10.1111/acer.15463 (PMC11629458; doi:10.1111/acer.15463)
Supplement: Supplementary file 1 — TABLES S1–S3. [file ACER-48-2303-s001.zip › 6039R2_Moirand_AcceptedSuppLegends_LD.docx]

**TABLE S1** Characteristics of patients attending the hepatology visit.

^1^ delay in days between first TE screening and the hepatologist visit.

^2^Cirrhosis clinical signs (firm liver, spider naevi)

^3^ Duration in years of at-risk alcohol use (more than 3 standard drinks per day).

³ Rhythm of alcohol consumption.

Abbreviations: WC, waist circumference in cm; BMI, body mass index in kg/m^2^.

Patients are considered as having hypertension or diabetes when treated.

**TABLE S2** Hepatic parameters of patients attending the hepatology visit.

AST, ALT, GGT, and PAL in IU/L; LSM in kPa. Bilirubin in µmole/liter
Normal levels: AST < 40 IU/L, ALT < 41 IU/L, PAL < 129 IU/L, GGT < 60 IU/L.
Patients are considered to have hyperlipemia when treated or having lipid results above laboratory normal range.

**TABLE S3** Evolution of alcohol consumption between the two visits.

¹Average alcohol consumption in the past 7 days (grams/day).

²Motivation for alcohol cessation using a VAS from 0 to 10.

³Craving for alcohol consumption using a VAS from 0 to 10.
