# Supplementary material for: Screening for compensated advanced chronic liver disease using transient elastography in outpatient addiction clinics
Source: Alcohol Clin Exp Res (Hoboken). 2024 Oct 13;48(12):2303–9. doi: 10.1111/acer.15463 (PMC11629458; doi:10.1111/acer.15463)
Supplement: Supplementary file 1 — TABLES S1–S3. [file ACER-48-2303-s001.zip › table_S1.docx]

| **Patients** | **Age** | **visit delay^1^** | **Cirrhosis signs^2^** | **WC** | **BMI** | **Exposure duration^3^** | **Rhythm^4^** | **Hypertension** | **Diabetes** |
| --- | --- | --- | --- | --- | --- | --- | --- | --- | --- |
| 1 | 68 | 23 | no | 96 | 26.6 |  | daily | no | yes |
| 2 | 56 | 42 | no | 113 | 30.9 | 39 | abstinent | no | yes |
| 3 | 55 | 26 | no | 110 | 30.9 | 33 | daily | yes | yes |
| 4 | 61 | 26 | no | 117 | 31 | 46 | daily | yes | no |
| 5 | 42 | 20 | no | 77 | 21.4 | 12 | daily | no | no |
| 6 | 48 | 21 | yes | 126 | 34.5 | 30 | occasionally | yes | no |
| 7 | 60 | 14 | no | 131 | 39 | 19 | irregularly | yes | yes |
| 8 | 62 | 21 | no | 85 | 22.5 | 45 | daily | no | no |
| 9 | 44 | 14 | no | 82 | 22.2 | 30 | daily | no | no |
