# Supplementary material for: Screening for compensated advanced chronic liver disease using transient elastography in outpatient addiction clinics
Source: Alcohol Clin Exp Res (Hoboken). 2024 Oct 13;48(12):2303–9. doi: 10.1111/acer.15463 (PMC11629458; doi:10.1111/acer.15463)
Supplement: Supplementary file 1 — TABLES S1–S3. [file ACER-48-2303-s001.zip › table_S2.docx]

| **Patients** | **Dyslipidemia** | **Platelets** | **bilirubin** | **ASAT** | **ALAT** | **GGT** | **PAL** | **First TE** | **Second TE** | **cACLD** |
| --- | --- | --- | --- | --- | --- | --- | --- | --- | --- | --- |
| 1 | **Yes** | >150 | 6.9 | 22 | 29 | 73 | 45 | 8.3 | 10.9 | yes |
| 2 | **yes** | >150 | 7.5 | 15 | 19 | 41 | 106 | 21.2 | 14 | yes |
| 3 | **no** | >150 | 5.8 | 65 | 92 | 149 | 125 | 16.9 | 16.3 | yes |
| 4 | **yes** | <150 | 5.4 | 33 | 25 | 84 | 178 | 52.5 | 55 | yes |
| 5 | **yes** | >150 | 9 | 18 | 14 | 25 | 83 | 43.6 | 3.9 | no |
| 6 | **yes** | >150 | 19 | 20 | 28 | 58 | 98 | 8.3 | 7.6 | no |
| 7 | **yes** | >150 | 4.3 | 32 | 43 | 62 | 128 | 11.9 | 18.3 | yes |
| 8 | **no** | >150 | 6.7 | 27 | 14 | 57 | 21 | 8.3 | 7.1 | no |
| 9 | **no** | >150 |  |  |  |  |  | 8.3 | 7.1 | no |
