# Supplementary material for: Screening for compensated advanced chronic liver disease using transient elastography in outpatient addiction clinics
Source: Alcohol Clin Exp Res (Hoboken). 2024 Oct 13;48(12):2303–9. doi: 10.1111/acer.15463 (PMC11629458; doi:10.1111/acer.15463)
Supplement: Supplementary file 1 — TABLES S1–S3. [file ACER-48-2303-s001.zip › table_S3.docx]

| **Patients** | **Alcohol consumption¹** | | **Motivation²** | | **Craving³** | |
| --- | --- | --- | --- | --- | --- | --- |
|  | **Screening** | **Consultation** | **Screening** | **Consultation** | **Screening** | **Consultation** |
| 1 | 40 | 50 | 3 | 0 | 0 | 0 |
| 2 | 0 | 0 | 10 | 10 | 0 | 0 |
| 3 | 20 |  | 8 |  | 4 |  |
| 4 | 20 | 20 | 8 | 9 | 2 | 2 |
| 5 | 30 | 140 | 9 | 9 | 5 | 5 |
| 6 | 90 | 30 | 8 | 8.5 | 8 | 4 |
| 7 | 0 | 0 | 10 | 10 | 3 | 3 |
| 8 | 30 | 20 | 10 | 7 | 10 | 8 |
| 9 | 30 |  | 5 |  | 10 |  |
| **median** | **30** | **30** | **8** | **9** | **4** | **3** |
